# Purple Sweet Potato Polyphenols Differentially Influence the Microbial Composition Depending on the Fermentability of Dietary Fiber in a Mixed Culture of Swine Fecal Bacteria

**DOI:** 10.3390/nu11071495

**Published:** 2019-06-30

**Authors:** Aldrine Kilua, Riri Nomata, Ryuji Nagata, Naoki Fukuma, Kenichiro Shimada, Kyu-Ho Han, Michihiro Fukushima

**Affiliations:** 1 Department of Life and Food Science, Obihiro University of Agriculture and Veterinary Medicine, Obihiro, Hokkaido 080-8555, Japan; 2 The United Graduate School of Agricultural Sciences, Iwate University, 3-18-8 Ueda, Morioka 020-8550, Japan; 3 Research Center for Global Agromedicine, Obihiro University of Agriculture and Veterinary Medicine, Obihiro, Hokkaido 080-8555, Japan

**Keywords:** anthocyanins, purple sweet potato, fermentation, inulin, cellulose, microbiota, polyphenols

## Abstract

The prevalence of many chronic diseases which have been associated with poor nutrition may be reduced by the positive modulation of colonic microbiota. In this study, we assess the effects of purple sweet potato polyphenols (PSP) in a mixed culture of swine fecal bacteria during in vitro colonic fermentation using pig colonic digest. Jar fermenters were used to conduct a small scale in vitro colonic fermentation experiments under the anaerobic condition for 48 h. Jar fermenters were assigned to one of the following groups: Cellulose, cellulose + PSP, inulin, and inulin + PSP. The present study revealed that the polyphenolic content of purple sweet potato could modulate the colonic microbiota by differentially increasing the population of beneficial bacteria and decreasing the pathogenic bacteria depending on cellulose and inulin. Accordingly, PSP might be a material conducive for improving the conditions for the fermentation of partly-fermentable dietary fiber. Besides, PSP was also responsible for the drastic reduction of putrefactive products, especially *p*-cresol to a significant level. Our results suggest that PSP could alter the microbial composition depending upon the fermentability of dietary fiber and has the potential to maintain a stable and healthy colonic environment that will ultimately alleviate chronic diseases development and confer health benefits to the host.

## 1. Introduction

The prevalence of chronic diseases (cardiovascular diseases, heart disease, stroke, cancer, chronic respiratory diseases, and type 2 diabetes) afflicting populations from both developed and developing countries is particularly associated with poor nutrition and physical inactivity [1,2]. Apart from non-dietary attributes, the dietary pattern is an important factor in the development and subsequent deleterious effects of chronic diseases [3,4]. Although high intake of energy-dense food and physical inactivity appear to be the primary causes associated with the development of chronic diseases, it is now confirmed that gut microbiota plays a critical role in relation to chronic diseases development [5].

Gut microbiota contains more than 30,000 different species of bacteria and considered an organ of its own, although dominated by Bacteroidetes and Firmicutes phyla [6,7]. Although diverse and complex, it can be easily influenced by the diet [8]. For these reasons, an imbalance in the composition of a diet has been linked to inflammation and inflammatory bowel disease [9,10]. Hence, modulation of colonic microbiota might be an ideal solution to alleviate chronic diseases development. 

Inulin, for instance, is a fermentable dietary fiber and regarded as a prebiotic compound due to its prebiotic efficacy [11]. It is indigestible in the small intestine, but fermented in the colon and modulates the colonic microbiota by selectively increasing the population of beneficial bacteria while inhibiting the pathogenic ones [12]. Conversely, cellulose is generally poorly fermented by the gut microbiota, but their presence is of importance in that it reduces the time available for colonic bacterial fermentation of non-digested materials. That is, its physicochemical characteristics and water-holding capacity increases bulking and forces the material through the gut faster and consequently reduces the time for bacterial fermentation [13]. 

Besides, polyphenols are groups of compounds found in various plants, fruits and vegetables [14], and has come under intense instigation recently for their ameliorative attributes towards cancer, cardiovascular diseases and antimicrobial activity [15,16,17]. Depending upon the chemical structure of phenolic moiety and any attached chemical groups, polyphenols can either absorb in the small intestine or reaches the colon for microbial attack and consequently reduces the risk of chronic diseases caused by oxidative stress. Thus, a thorough examination of the effect of polyphenol on various diseases has been carried out. 

Purple sweet potato is one of the nutritional vegetables consumed worldwide. It has a high total phenolics and antioxidant capacity [18,19]. Besides, it has high anthocyanin content because of its color attributes. The predominant anthocyanins in purple sweet potato are peonidin- and cyanidin-glucoside and they are highly acylated with caffeic, ferulic, and/or hydroxybenzoic acids [18]. Purple sweet potato polyphenols from different varieties have been studied previously for their effects in relation to absorption, antioxidant, anti-inflammatory, anti-tumor and hypoglycemic effect [19,20,21]. Although these studies focused on health implications associated with polyphenols, however, there are remarkably very few studies addressing the effects on colonic microbiota. Therefore, the aim of the current study is to assess the effects of PSP in combination with cellulose (partly fermentable dietary fiber) or inulin (fermentable dietary fiber) in a mixed culture of swine fecal bacteria during in vitro colonic fermentation.

## 2. Materials and Methods 

### 2.1. Preparation and Determination of Sweet Potato Polyphenols

Preparations of purple sweet potato polyphenols [PSP] extract were done according to Han et al. [22] with slight modifications. Briefly, 50 g of the powder of purple sweet potato (*Ipomoea batatas* cv. Ayamurasaki) was subjected to 70% acetone and sonicate for 20 min three times until almost all colored pigments were extracted. The suspensions were filtered and centrifuged at 14,600 × *g* and 4 °C for 30 min. The acetone was removed by using rotary evaporator at 35 °C, and the pigment extract was dissolved with distilled water and applied directly to pretreated Diaion HP-20 resin (Nippon Rensui Co., Tokyo, Japan) column. Three types of solutions were consecutively added to the elution column, namely, water, 20% ethanol, and 80% acetone. The eluate contains 1.54%, 5.06%, and 93.4% PSP respectively. Water and ethanol fractions of PSP were not used for the current study as it contained impurities like free sugars, proteins, salts, and lower molecular weight polyphenol fractions. The pooled eluate from 80% acetone was evaporated using rotary evaporator to remove acetone at 35 °C, and the concentrate was dissolved in distilled water and vacuum filtered to remove bacteria using 0.22 µm sterile disposable filter (Nalgene rapid-flow disposable filter units with PES membrane, Thermo Fisher Scientific, Tokyo, Japan) and stored for in vitro studies. The total polyphenol content of purple sweet potato extract was determined according to the Folin–Ciocalteu method [23]. Briefly, 0.5 mL of the diluted sample was mixed with 5 mL of NaCO_3_ and then shaken. After 5 min, 0.5 mL of Folin-Ciocalteu reagent was added to the mixture and vigorously shaken. After 30 min, the absorbance was measured at 750 nm using Shimadzu 1600-UV spectrophotometer. The total phenolic content of the extract was approximately 79% as gallic acid equivalents. 

### 2.2. Feces and In Vitro Fermentation

In vitro fermentation was conducted under an anaerobic condition with a mixture of fresh pig feces collected from Tokachi Hills farm in Obihiro. The pig’s feces were collected directly from the anus of three pigs and inserted into an anaerobic double zipper plastic bag containing AnaeroPack-Anaero (Mitsubishi Gas Chemical, Tokyo, Japan) without exposure to air. The pig feces were collected on site on the day of the experiment. Fecal slurry (10× dilution) was prepared by homogenizing equal amounts from three pigs in a stomacher (Exnizer 400, Organo Co., Tokyo, Japan) and filtered through a stomacher bag (Eiken Chemical Co., Ltd., Tokyo, Japan) filled with 0.85% NaCl solution and used as inocula. The pH-controlled jar fermenters (220 mL working volume, Able & Biott, Tokyo, Japan) were inoculated with the fecal slurry to give a final concentration of 2.0% (*v*/*v*). Prior to samples treatment, a pre-incubation period of 12 h was scheduled to stabilize the growth of microbes in the fermenters. After pre-incubation, one of the following samples was added to each fermenter, 3% cellulose (CEL), 3% cellulose + 0.16% PSP (CELP), 3% inulin (INU), and 3% inulin + 0.16% PSP (INUP). We used PSP equivalent per jar based on a study by Nagata et al. [24]. A final concentration of 0.8% (*w*/*v*) of the autoclaved basal nutrient broth (Difco, Sparks, MD, USA) was added to each fermenter. The fermentation design was anaerobically maintained under CO_2_ gas at 37 °C for 48 h at a lower pH limit of 5.50. At 0, 6, 12, 24 and 48 h time points, pH was recorded and aliquots were collected in 2 mL tubes and stored at −80 °C for further analysis. This experimental design was reviewed and approved by the Animal Experiment Committee of Obihiro University of Agriculture and Veterinary Medicine (no. 18–32).

### 2.3. Bacterial Analysis

Bacterial populations were analyzed using selective media by plate count method and were incubated at 37 °C using AnaeroPack-Anaero (Mitsubishi Gas Chemical) in a sealed anaerobic container.

### 2.4. DNA Extraction and 16S Ribosomal RNA (16S rRNA) Gene Sequences

Bacterial DNA was extracted from 48 h samples (non-diluted) using a modified phenol-free repeated bead beating plus column (RBB + C) method described by Yu & Morrison [25]. After extraction, the genomic DNA was purified via sequential digestions with RNase and proteinase K using QIAamp columns from the QIAamp DNA tool Mini kit (QIAGEN, Valencia, CA, USA). The concentration of the extracted DNA community was measured by Nanodrop 2000c spectrophotometer (Thermo Fisher Scientific) and was adjusted to 5 ng/µL with Tris-EDTA buffer. V3-V4 variable regions of the 16S rRNA gene were amplified using the following bacterial overhang adapters and universal primers in the first stage of polymerase chain reaction (PCR), forward primer (5’-TCG TCG GCA GCG TCA GAT GTG TAT AAG AGA CAG CCT ACG GGN GGC WGC AG-3’) and the reverse primer (5’-GTC TCG TGG GCT CGG AGA TGT GTA TAA GAG ACA GGA TTA CHV GGG TAT CTA ATC C-3’). In the second stage PCR, Illumina sequencing adapters and dual index barcodes were added to the amplicons using Nextera^®^ XT Index Kit (Illumina Inc., San Diego, CA, USA). After quantification of PCR products using QuantusTM fluorometer (Quantifluor^®^ dsDNA System, Promega, Madison, WI, USA), the successful PCR products were pooled in one tube with equal volumes and subjected to paired-end sequencing by Illumina MiSeq System (Illumina Inc.). The analysis of retrieved raw 16S rRNA gene sequences was conducted according to Warren et al. [26] and the generated biome table was normalized using an equal subsampling size of 6727 sequences. Calculation of distances between bacterial communities in different samples by the weighted UniFrac distance metric and preparation of principal coordinate analysis (PCoA) plot were conducted in QIIME [27]. Calypso version 8.84 [28] was used to visualize α-diversity (observed species and Shannon index) and hierarchical clustering plots at the phylum level. 

### 2.5. Short-Chain Fatty Acid (SCFA) Analysis

Aliquots from fermenters were centrifuged (9,200 × *g* and 4 °C for 15 min) and the supernatants were filtered with a 1 mL syringe after 0.5 N HClO_4_ was added to deproteinize the filtrates. The filtrates were subjected to HPLC in a Shimadzu LC-10AD (Kyoto, Japan) equipped with an ST3-R post-column. The analytical conditions were as follows: Column, RSpak KC-811 (8.0 mm × 300 mm, Shodex, Tokyo, Japan), eluent and flow rate, 2 mM perchloric acid at 1.0 mL/min, column temperature, 47 °C, reaction reagent and flow rate, ST3-R (10× diluted, Shodex) at 0.5 mL/min, UV detector wavelength, 430 nm. 

### 2.6. Measurement of Putrefactive Products

The concentration of ammonium nitrogen was measured using a commercially available kit (Wako Pure Chemical Industry, Ltd., Tokyo, Japan) according to the manufacturer’s instructions. The concentration of *p*-cresol at 48 h was measured according to Ikeda et al. [29] as follows: In a tube of a 0.1 mL sample, was added acetonitrile, anhydrous sodium sulfate, and acetonitrile-saturated hexane. The mixture was then shaken vigorously using a mixer for 2 min and centrifuged (1500 × *g*, 5 min). Acetonitrile-saturated hexane was added to the middle of acetonitrile phase in a new tube, and the mixture was shaken vigorously for 1 min and centrifuged (1500 × *g*, 5 min). The lower acetonitrile phase was used for analysis after filtration with a syringe filter. A CTO-10A Shimadzu column (150 × 4.6 mm, Shimadzu Co, Tokyo, Japan) was used in a column oven at 40 °C. The mobile phase, consisting of acetonitrile/water (30:70, *v*/*v*), was flowed at a constant flow rate of 1.0 mL/min. The UV detector wavelength was set at 280 nm.

### 2.7. Statistical Analysis

All data are presented as a mean and standard error (SE). The in vitro fermentation was conducted in a block design with replicates of five. Two-way ANOVA was performed to assess the effect of fiber (cellulose and inulin), PSP, and their interaction. Differences of *p* < 0.05 was taken to be statistically significant. If the variance was observed in the main effect of interaction, Tukey’s test was used for this comparison (*p* < 0.05). Analyses were performed using PASW Statistics 17.0 software (SPSS Institute, Armonk, NY, USA).

## 3. Results 

### 3.1. Gut Microbial Taxonomic Analysis 

**Figure 1:**
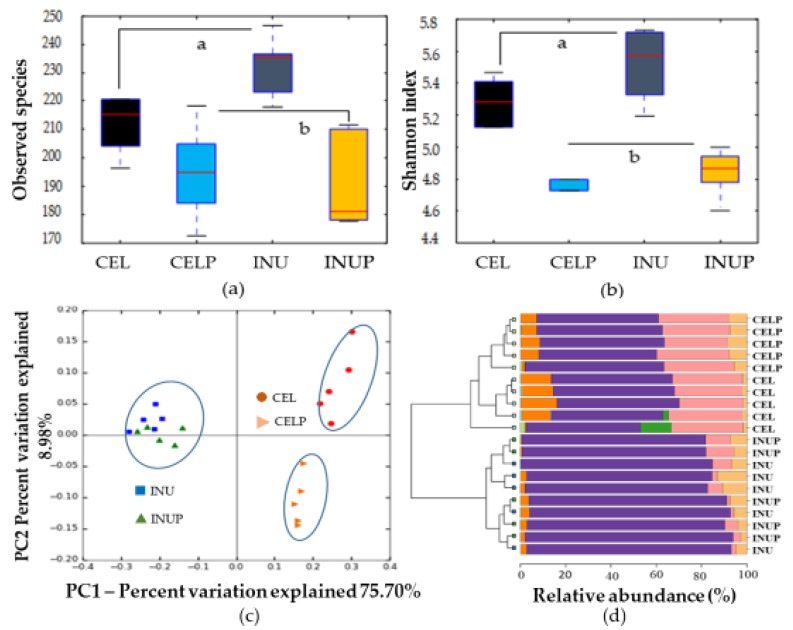
α-diversity (observed species (**a**) and Shannon diversity index (**b**)), β-diversity (**c**) and clustered bar-chart (**d**) comparisons of microbiota during in vitro colonic fermentation of 3% cellulose (CEL), 3% cellulose + 0.16% PSP (CELP), 3% inulin (INU), and 3% inulin + 0.16% PSP (INUP). Observed species (**a**) and Shannon diversity index (**b**) were compared by using the non-parametric Kruskal–Wallis rank sum test. Different letters are significant at *p* < 0.05.

After 48 h of in vitro fermentation, we assessed the treatments on microbial composition by analyzing the 48 h samples using 16S rRNA amplicon. The α-diversity (observed species (Figure 1a) and Shannon index (Figure 1b)) showed significant (*p* < 0.05) difference for the CEL and CELP groups compared with the INU and INUP groups. This highlights the differences in the fermentability between the CEL and INU groups. That is low and high fermentability, respectively. In order to evaluate the similarities and differences between groups, PCoA (β-diversity) was performed (Figure 1c). Four groups were separated into two distinct clusters along PC1 (75.7%), highlighting the dominant microbiota amongst the groups. Interestingly, the CELP group was distinctively separated along the PC2 axis. Accordingly, the clustered bar-chart at phyla level (Figure 1d) shows that the INU and INUP groups were clustered together while the CEL and CELP groups formed two distinctively separate clusters. 

**Figure 2:**
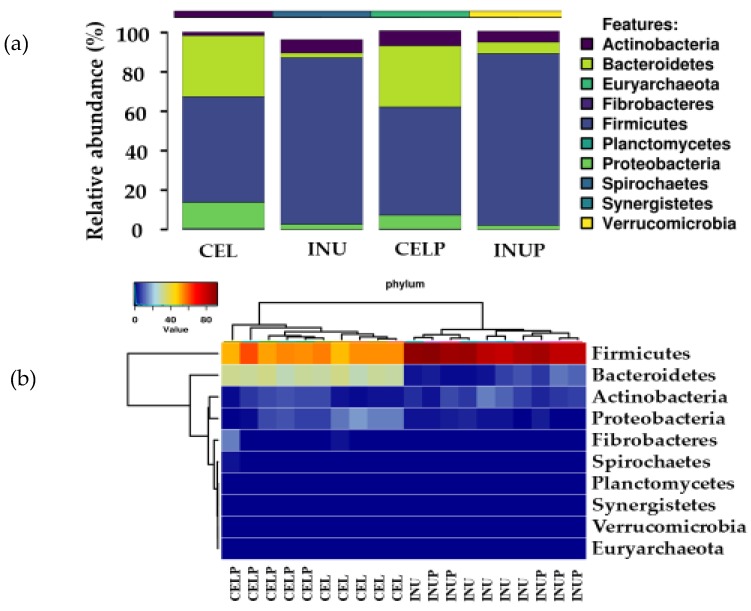
Relative abundances (**a**) and heat map (**b**) showing the predominant bacterial phyla of Firmicutes, Bacteroidetes and Actinobacteria during in vitro colonic fermentation of 3% cellulose (CEL), 3% cellulose + 0.16% PSP (CELP), 3% inulin (INU), and 3% inulin + 0.16% PSP (INUP).

We then determined which phyla were predominant, and found that the microbiota was dominated by Firmicutes and Bacteroidetes followed by Actinobacteria. Further, it was also observed that phylum enrichment was associated with the type of diet (Figure 2). Interestingly, we noticed that when PSP was combined with cellulose (CELP), it increases the relative abundance of Actinobacteria. Besides, the PSP depressed the abundance of phylum, Proteobacteria, and this effect was stronger in the CELP than in INUP group.

At the genus level, we observed that PSP partly modulated the microbial composition depending on the fermentability of dietary fiber (Table 1). For example, the relative abundance of *Prevotella* was increased when PSP was combined with inulin, but no effect was seen for the CELP group. PSP supplementation also increases the relative abundance of *Bifidobacterium* in the CELP group but decreased in the INUP groups. Likewise, some bacterial genera belonging to Firmicutes were also modulated by PSP supplementation depending on the fermentability of dietary fiber. For instance, the relative abundance of *Clostridium* in the CELP group was less compared with the CEL group, although no significant effect can be seen between the INU and INUP groups. This effect was attributed to the interactive effect of cellulose and PSP. Interestingly, the relative abundance of *Lactobacillus* was enhanced in both CELP and INUP groups respectively due to the effect of PSP supplementation. Similarly, PSP supplementation also increased the population of *Lactobacillus* in the CELP and INUP groups (Table 1). In the relative abundance of *Sharpea*, PSP supplementation suppressed the abundance in the CELP group, while the INUP group was kept at a lower level with the positive control group (INU). The relative abundance of *Coprococcus* was increased in the INUP group, and *Bulleidia* was increased in both CELP and INUP groups compared with their single supplement of the CEL and INU groups due to the attribution of PSP and the interaction effect respectively. Interestingly, the interaction between cellulose and PSP decreased the relative abundance of *Acidaminococcus* in the CELP group, without any effect, or the same as the positive control for the INUP group.

**Figure 3:**
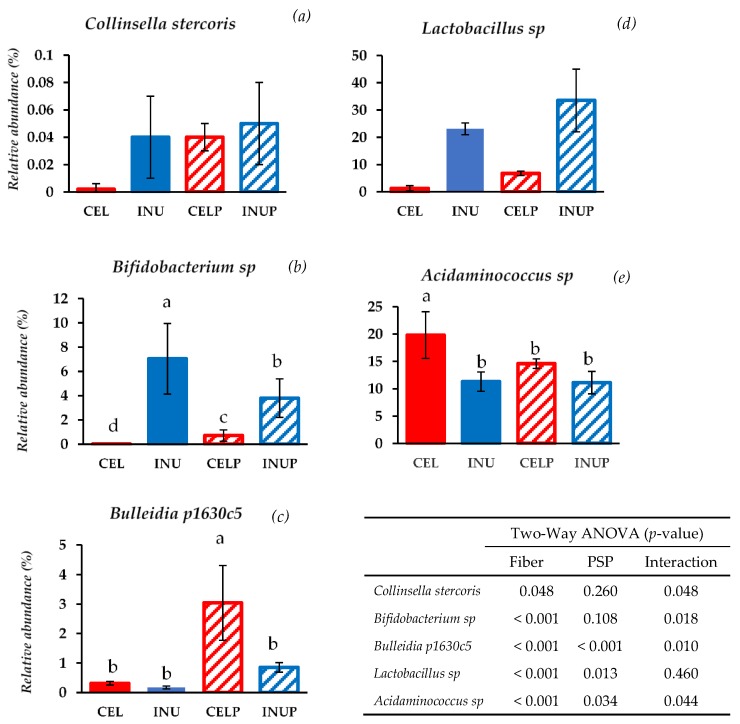
Relative abundances at species level: *Collinsella stercoris* (**a**), *Bifidobacterium* sp. (**b**)*, Bulleidia p1630c5 (***c**), * Lactobacillus* sp. (**d**), and *Acidaminococcus* sp. (**e**) after 48 h of treatment during in vitro colonic fermentation of 3% cellulose (CEL), 3% cellulose + 0.16% PSP (CELP), 3% inulin (INU), and 3% inulin + 0.16% PSP (INUP). Statistical significance amongst the groups was determined by two-way ANOVA analysis to assess the effect of fiber (CEL and INU), PSP and their interaction. *P* < 0.05 was considered to be statistically significant. If the variance was observed in the main effect of the interaction, Tukey’s test was used for this comparison (*p* < 0.05).

At the species level, bacterial species belonging to Actinobacteria were modulated by PSP supplementation such as *Collinsella stercoris* and *Bifidobacterium* sp. (Figure 3). When PSP was combined with cellulose (CELP), it increases the relative abundance of *Collinsella stercoris* to the level of the INU and INUP groups. It is interesting to note that *Bifidobacterium* sp. was more abundant when PSP was combined with cellulose (CELP) but decreases when supplemented with inulin (INUP), the attribution due to the interaction and the effect of PSP respectively. With regards to species belonging to Firmicutes, *Bulleidia p1630c5* and *Lactobacillus* sp. increases in both CELP and INUP groups, particularly due to the interaction effect and the effect attributed to PSP. Interestingly, when PSP was combined with cellulose (CELP), the increase in *Acidaminococcus* sp. was thwarted. However, when combined with inulin (INUP), no significant changes to that of INU group but improves its effect in reducing *Acidaminococcus* sp. This is particularly associated with PSP and its interaction with inulin.

### 3.2. SCFA Concentration in Fermenters

Table 2 shows the cumulative concentrations of SCFA in the fermenters during in vitro colonic fermentation. In our study, although the INU and INUP groups were significantly higher than the CEL and CELP groups, we observed that PSP had neither a negative nor positive impact on SCFA production. The differences in the cumulative productions of SCFA was associated with the fermentability of dietary fiber (CEL and INU).

### 3.3. pH in Fermenters

**Figure 4:**
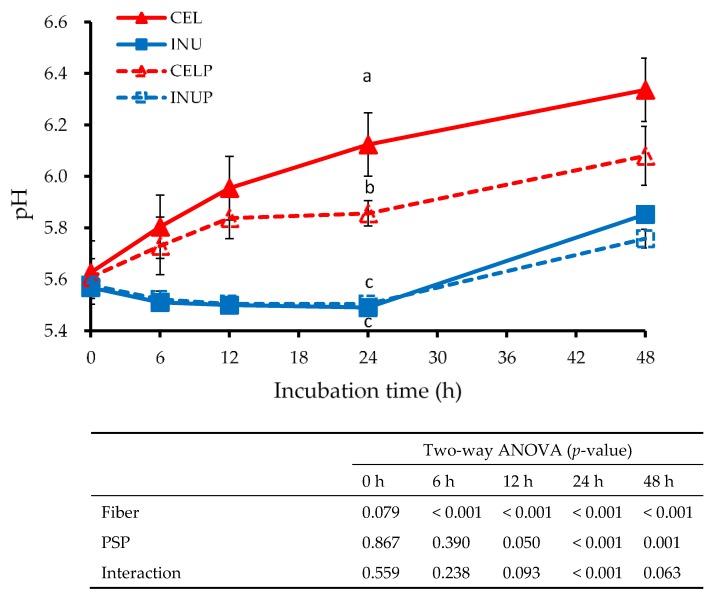
The pH values for each sample treatments during in vitro colonic fermentation of 3% cellulose (CEL), 3% cellulose + 0.16% PSP (CELP), 3% inulin (INU), and 3% inulin + 0.16% PSP (INUP). Values are reported as mean and standard error (*n* = 5). Two-way ANOVA was performed to assess the effect of fiber (cellulose and inulin), PSP, and their interaction. If the variance was observed in the main effect of the interaction, Tukey’s test was used for this comparison. Mean values at the same time point designated by different letters (a–c) are significantly different (*p* < 0.05).

Figure 4 shows the pH value at different time points for each treatment during fermentation. Throughout the study, the INU and INUP groups were significantly (*p* < 0.05) lower than the CEL and CELP groups, although the CELP group was significantly lower than the CEL group. The effect due to fiber was observed throughout the study. In addition, we also observed at 12 to 48 h time points that the reduction in the pH value was attributed to PSP. Besides, the interaction effect was also noticed at the 24 h time point. 

### 3.4. Putrefactive Products in Fermenters

**Figure 5:**
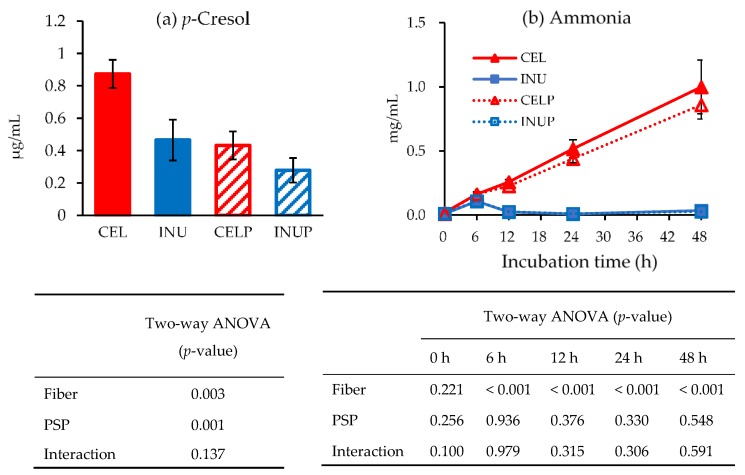
*p*-Cresol concentration (**a**) and ammonia production (**b**) for each treatment during in vitro colonic fermentation of 3% cellulose (CEL), 3% cellulose + 0.16% PSP (CELP), 3% inulin (INU) and 3% inulin + 0.16% (INUP). Values are reported as mean and standard error (*n* = 5). Two-way ANOVA was performed to assess the effect of fiber (cellulose and inulin), PSP, and their interaction. Differences of *p* < 0.05 was taken to be statistically significant.

Figure 5 shows the concentrations of *p*-cresol (a) and ammonia (b) amongst the groups during in vitro colonic fermentation. At the end of 48 h, we observed that the effect associated with PSP caused a drastic decrease in the concentration of *p*-cresol (*p* < 0.05), which was reflected in PSP combination groups (INUP and CELP). Although the ammonia concentrations of both INU and INUP groups were significantly (*p* < 0.05) lower than the CEL and CELP groups, respectively, throughout the study, PSP did not have any significant effect. The effect was associated with dietary fiber.

## 4. Discussion

There are remarkably very few studies on PSP addressing its health implications on colonic microbiota. Previously, studies have been conducted on PSP to substantiate its beneficial effects in relation to chronic disease prevention, with an approach different from the current study [20,21]. In the present study, we are particularly applied an in vitro approach to assess the effects of PSP in association with a poorly fermentable dietary fiber (cellulose) and fermentable dietary fiber (inulin) in a mixed culture of swine fecal bacteria. We applied an in vitro approach in an attempt to address that PSP has the potential to alter gut microbiota, and by affecting the total number of beneficial bacteria, may confer positive health benefits. We used swine feces because of the similarities in the digestive systems and the intestinal ecology between pigs and humans [30]. 

In the present study, we observed that the microbial composition was affected by the treatment types, particularly in response to the type of dietary fiber (cellulose or inulin) in terms of their fermentability. Our genomic analysis based on 48 h samples revealed an altered microbial community. Schloss et al. [31] stated that an estimate of community’s richness is based on observed species index, while Shannon index is an estimate of species diversity. In our study, we observed that although the fermentability of cellulose and inulin were responsible for the community’s richness and species diversity, PSP supplementation also distorted the community’s richness and diversity. For example, the increase in species richness and diversity in the CELP group was attributed to PSP. Frolinger et al. [32] reported that preparations from grape polyphenols demonstrated a distinct alteration in microbial diversity. Besides, our study also revealed that the CELP group was separated along the PC2 axis, which in fact, reflects that microbial composition was affected by PSP supplementation. This was confirmed by the fact that the CEL and CELP groups formed two distinctively separated clusters. Consistently, Espley et al. [33] highlighted that supplementation with apple anthocyanin affects species richness and diversity. Obviously, the fermentability of cellulose and inulin were responsible for the distinct differences in the microbial community. Besides, we noticed that PSP improved the fermentability condition for cellulose. This phenomenon could be associated with the upregulation of certain bacterial enzymes by PSP, as a result of the combination effect.

Besides, the microbial composition of the phylum was also modulated by PSP depending on which dietary fiber PSP was associated with. For instance, in the phylum of Actinobacteria, when cellulose was combined with PSP (CELP), it increased the relative abundance but reduced when combined with inulin (INUP). This shows that bacterial proliferation can differentially be enhanced or thwarted by PSP depended on dietary fiber. In the genus level, our result indicated that supplementing PSP with inulin increased the relative abundance of *Prevotella*. Kovatcheva-Datchary et al. [34] highlighted that the proliferation of *Prevotella* is associated with a diet specific, species and/or strains of *Prevotella* present and other microbe-microbe interactions. In our study, it could be related to PSP supplemented diet. In addition, while the relative abundance of *Bifidobacterium* decreased in the INUP group, it increased in the CELP group, an attribution that PSP can upregulate certain beneficial bacterial enzymes that were suppressed in less fermentable dietary fiber. Further, most of the bacterial genus belonging to Firmicutes were affected by PSP supplementation. *Clostridium,* for instance, was reduced in the CELP group due to the interaction effect, and the reduction of *Clostridium* is a positive response because *Clostridium* is a pathogen responsible for many gastrointestinal illnesses [35]. One of the beneficial butyrate-producing bacteria associated with colonic health is *Eubacterium*. In the current study, it increased in both CELP and INUP groups because of the interaction between dietary fiber and PSP. This could possibly due to the use of ammonia as a nitrogen source, which was also reflected in the decrease in ammonia production in both groups. Besides, *Coprococcus* can efficiently ferment dietary fiber and other complex carbohydrates to butyrate, the metabolite responsible for the inhibition of colonic inflammation and carcinogenesis [36]. In our study, we observed that the relative abundance increased in the INUP group and this increase was associated with PSP. This explains that PSP can differentially increase or decrease certain bacterial enzymes depending upon the fermentability of dietary fiber. In this case, bacterial enzymes responsible for butyrate production might be enhanced in the INUP group. Interestingly, the relative abundance of *Acidaminococcus* was suppressed when cellulose combined with PSP (CELP), while PSP enhanced the positive effect of inulin. The reduction in *Acidaminococcus* was correlated with a higher fiber diet during fermentation. When PSP was added, it positively affected both the combination groups. According to an in vitro study by Zhang et al. [37], anthocyanin from purple sweet potato can increase the growth of beneficial bacteria such as *Bifidobacterium* and *Lactobacillus* by the metabolism of polyphenols and their corresponding monomers. Consistently, the increase in *Lactobacillus* population in our study was attributed to PSP and the effect due to the interaction of fiber and PSP. Thus, the increments of the beneficial bacterial genus in our study might be associated with the metabolism of PSP, including their corresponding monomers. We also noticed that effect due to PSP was responsible for the proliferation and suppression of beneficial and potentially pathogenic bacterial species respectively. For instance, the increase in *Collinsella stercoris, Bulleidia p1630c5, Bifidobacterium* sp., and *Lactobacillus* sp., and the decrease in *Acidaminococcus* sp. in our study were attributed to both PSP and the interaction effect. Molan et al. [38] stated that the blackcurrant extract increases the counts of beneficial bacterial species while decreasing the counts of potentially harmful species. Besides, *β*-glucosidase and *β*-glucuronidase increases and decreases respectively. This could be at play in the current study. That is, the enzyme responsible for pathogenic bacterial growth was suppressed by anthocyanin contained in PSP.

Epidemiological studies have shown that SCFA (acetate, propionate, and butyrate) formation during colonic fermentation confers beneficial health properties to the host [39]. Acetate, being one of the most common and abundant SCFA in the human colon, is associated with the suppression of adipocyte lipolysis, and consequently mitigating against fatty liver induced deterioration in glucose homeostasis [40]. Likewise, propionate inhibits the expression of lipopolysaccharide (LPS)-induced cytokines, IL-6, and IL12 p40 in human mature dendritic cells [41]. Butyrate, on the other hand, is an important colonocytes energy source, and thus, inhibits histone deacetylation in acute myeloid leukemia [42]. In our study, PSP did not either have any positive or negative effects on SCFA production, although the INU and INUP groups were significantly higher than the CEL and CELP groups, the attribution due to the differences in the fermentability of cellulose and inulin. Colonic pH can be a marker of colonic health as well. That is, the reduction in pH suppressed the proliferation of pathogenic bacteria while increasing the number of beneficial bacterial counts [43]. In our study, we observed that PSP was also responsible for the reduction of colonic pH from 12 to 48 h time points. One reason could be due to the utilization of sugar moiety present in PSP by the microbiota and subsequently acidify the colonic environment. The other possibility could be the microbial conversion of dietary fiber and PSP might influence the production of organic acid, and consequently reduces the level of pH. Zhu et al. [44] reported that microbial conversion of polyphenols affects other colonic pathways and processes, such as the production of formic or lactic acid. Accordingly, Gibson and Roberfroid [45] stated that the acidic colonic environment promotes the growth of *Lactobacillus* and *Bifidobacterium*. In this study, one of the main reasons for the acidic colonic environment is the high fermentability of dietary fiber, inulin, that consequently increased the production of SCFA and subsequently acidify the colonic environment. 

Although pH may reduce pathogenic bacteria and promote beneficial bacteria, an increase in the putrefactive product like ammonia or *p*-cresol may pose some risk factors to the host. Any studies have indicated that high ammonia concentration in the colon may be potentially harmful to the host. Davila et al. [46] highlighted that an increase in ammonia concentration can affect the energy metabolism of colonic epithelial cells. In our study, PSP neither has any positive or negative effects on ammonia production, although the INU and INUP groups were significantly lower than the CEL and CELP groups. Conversely, the *p*-cresol level was suppressed by PSP. *p*-Cresol is an aromatic compound produced by gut microbiota during fermentation of L-tyrosine [47]. The higher concentration is associated with chronic diseases and liver failure [48,49]. In our study, PSP was associated with the drastic reduction of the *p*-cresol level. This could be due to the molecular structure in PSP that might suppress the production of *p*-cresol, or, the enzyme activation responsible for *p*-cresol production was inhibited by PSP.

## 5. Conclusions

The present study demonstrates that the polyphenolic content of purple sweet potato can modulate the microbial composition by differentially proliferating and inhibiting the beneficial and pathogenic bacterial composition respectively depending on its association with fermentable and non-fermentable dietary fiber. Accordingly, PSP might be a material conducive for improving the conditions for the fermentation of non-fermentable dietary fiber. Besides, the drastic reduction of putrefactive products, especially *p*-cresol to a significant level was attributed to PSP. Further in vivo studies are needed to elucidate on polyphenolic compounds responsible for alleviating the risk of developing chronic diseases and enzymes responsible for the suppression and proliferation of bacteria. Although our study highlighted the positive impact PSP have on colonic microbiota, however, it is difficult to conclude with human settings because of the use of pig feces of which further research is warranted.

## Figures and Tables

**Table 1:** Changes in bacteria populations and relative abundance of genus during in vitro colonic fermentation.

	Groups				Two-Way ANOVA (*p*-Value)		
	CEL	INU	CELP	INUP	Fiber	PSP	Interaction
Anaerobes (log_10_ CFU mL^−1^)	8.20 ± 0.10	8.75 ± 0.10	8.70 ± 0.10	8.90 ± 0.10	0.001	0.003	0.080
*Lactobacillus* (log_10_ CFU mL^−1^)	6.57 ± 0.30 ^b^	7.25 ± 0.20 ^ab^	7.17 ± 0.10 ^ab^	7.86 ± 0.10 ^a^	0.002	0.005	0.048
Genus (Relative abundance, %)							
*Bacteroides*	1.74 ± 0.30	20.2 ± 1.6	3.79 ± 1.20	16.9 ± 6.5	<0.001	0.409	0.302
*Prevotella*	20.0 ± 2.4	3.64 ± 2.90	20.0 ± 2.4	6.39 ± 4.70	<0.001	0.008	0.367
*Bifidobacterium*	0.01 ± 0.01	7.03 ± 2.90	0.71 ± 0.50	3.78 ± 1.60	0.002	0.072	0.071
*Clostridium*	2.18 ± 1.20 ^a^	0.29 ± 0.10 ^b^	1.61 ± 0.70 ^a^	0.33 ± 0.20 ^b^	<0.001	0.108	0.018
*Lactobacillus*	1.27 ± 0.90	23.1 ± 2.1	6.76 ± 0.80	33.5 ± 11.0	<0.001	0.039	0.334
*Sharpea*	2.01 ± 1.50	0.02 ± 0.01	0.62 ± 0.60	0.02 ± 0.03	<0.001	0.354	0.356
*Coprococcus*	0.01 ± 0.01	0.02 ± 0.01	0.01 ± 0.01	0.04 ± 0.03	0.021	0.035	0.219
*Bulleidia*	0.82 ± 0.50 ^b^	0.33 ± 0.10 ^b^	3.42 ± 1.30 ^a^	1.06 ± 0.10 ^a^	<0.001	<0.001	0.010
*Acidaminococcus*	19.8 ± 4.3 ^a^	11.3 ± 1.8 ^b^	14.6 ± 0.9 ^b^	11.1 ± 2.1 ^b^	<0.001	0.034	0.044

Values are reported as mean and standard error (*n* = 5). Two-way ANOVA was performed to assess the effect of fiber (cellulose and inulin), PSP, and their interaction. Differences of *p* < 0.05 was taken to be statistically significant. If the variance was observed in the main effect of the interaction, Tukey’s test was used for this comparison. Mean values designated by different letters (a–b) amongst the groups are significantly different (*p* < 0.05).

**Table 2:** Changes in short chain fatty acid (SCFA) during in vitro fecal fermentation.

	Incubation Time (h)	CEL	INU	CELP	INUP	Two-Way ANOVA (*p*-Value)		
		µmol mL^−1^				Fiber	PSP	Interaction
Acetate	0	1.92 ± 2.00	2.00 ± 2.00	1.94 ± 0.22	1.94 ± 0.19	0.840	0.935	0.865
	6	4.54 ± 0.70	9.00 ± 1.50	5.68 ± 0.60	9.36 ± 1.50	0.003	0.529	0.744
	12	8.30 ± 0.70	34.2 ± 10.9	10.7 ± 0.4	31.6 ± 10.5	0.007	0.992	0.748
	24	12.0 ± 1.6	126 ± 20	16.6 ± 1.0	107 ± 25	<0.001	0.672	0.482
	48	32.8 ± 9.6	200 ± 18	32.9 ± 4.4	182 ± 27	<0.001	0.591	0.588
Propionate	0	0.57 ± 0.06	0.63 ± 0.07	0.54 ± 0.11	0.68 ± 0.11	0.255	0.935	0.664
	6	1.97 ± 0.92	2.55 ± 1.86	1.90 ± 1.01	2.60 ± 1.77	0.660	0.992	0.967
	12	3.97 ± 0.76	21.2 ± 12.5	5.10 ± 0.30	20.0 ± 13.3	0.097	0.995	0.898
	24	5.56 ± 0.85	83.8 ± 26.2	6.50 ± 0.60	65.5 ± 29.1	0.003	0.663	0.629
	48	13.5 ± 4.1	178 ± 12	12.6 ± 1.4	150 ± 23	<0.001	0.290	0.319
*n*-Butyrate	0	0.11 ± 0.05	0.08 ± 0.03	0.11 ± 0.05	0.07 ± 0.04	0.396	0.902	0.975
	6	0.57 ± 0.13	1.06 ± 0.19	0.70 ± 0.13	1.00 ± 0.20	0.017	0.846	0.789
	12	1.68 ± 0.27	2.25 ± 0.22	2.00 ± 0.18	1.88 ± 0.09	0.276	0.903	0.111
	24	3.07 ± 0.30	3.81 ± 0.34	3.29 ± 0.11	3.43 ± 0.28	0.127	0.754	0.288
	48	5.33 ± 0.92	10.2 ± 1.3	5.16 ± 0.33	9.55 ± 2.39	0.005	0.770	0.861
Total SCFA	0	2.60 ± 0.22	2.71 ± 0.30	2.58 ± 0.36	2.70 ± 0.31	0.722	0.960	0.994
	6	7.10 ± 1.70	12.6 ± 3.2	8.30 ± 1.70	13.0 ± 3.2	0.062	0.760	0.887
	12	14.0 ± 1.7	57.6 ± 22.8	17.8 ± 0.8	53.5 ± 24.1	0.030	0.992	0.812
	24	20.7 ± 2.5	213 ± 46	26.4 ± 1.4	176 ± 53	<0.001	0.661	0.549
	48	51.6 ± 14.6	388 ± 31	50.6 ± 6.1	341 ± 52	<0.001	0.447	0.465

Values of short chain fatty acids for each sample treatments during in vitro colonic fermentation of 3% cellulose (CEL), 3% cellulose + 0.16% PSP (CELP), 3% inulin (INU) and 3% inulin + 0.16% PSP (INUP). Values are reported as mean and standard error (*n* = 5). Two-way ANOVA was performed to assess the effect of fiber (cellulose and inulin), PSP, and their interaction.

## References

[1] Drewnowski A., Popkin B.M. (1997). The nutrition transition: New trends in the global diet. Nutr. Rev..

[2] Ferro-Luzzi A., Martino L. (1996). Obesity and physical activity. Ciba Found. Symp..

[3] Van Dam R.M., Rimm E.B., Willett W.C., Stampfer M.J., Hu F.B. (2002). Dietary patterns and risk for type 2 diabetes mellitus in U.S. men. Ann. Intern. Med..

[4] Kerver J.M., Yang E.J., Bianchi L., Song W.O. (2003). Dietary patterns associated with risk factors for cardiovascular disease in healthy US adults. Am. J. Clin. Nutr..

[5] Kobyliak N., Conte C., Cammarota G., Haley A.P., Styriak I., Gaspar L., Fusek J., Rodrigo L., Kruzliak P. (2016). Probiotics in prevention and treatment of obesity: A critical view. Nutr. Metab..

[6] Xenoulis P.G., Palculict B., Allenspach K., Steiner J.M., Van House A.M., Suchodolski J.S. (2008). Molecular-phylogenetic characterization of microbial communities’ imbalances in the small intestine of dogs with inflammatory bowel disease. FEMS Microbiol. Ecol..

[7] Eckburg P.B., Bik E.M., Bernstein C.N., Purdom E., Dethlefsen L., Sargent M., Gill S.R., Nelson K.E., Relman D.A. (2005). Diversity of the human intestinal microbial flora. Science.

[8] Graf D., Di Cagno R., Fåk F., Flint H.J., Nyman M., Saarela M., Watzl B. (2015). Contribution of diet to the composition of the human gut microbiota. Microb. Ecol. Health Dis..

[9] Dicksved J., Halfvarson J., Rosenquist M., Jarnerot G., Tysk C., Apajalahti J., Engstrand L., Jansson J.K. (2008). Molecular analysis of the gut microbiota of identical twins with Crohn’s disease. ISME J..

[10] Frank D.N., St Amand A.L., Feldman R.A., Boedeker E.C., Harpaz N., Pace N.R. (2007). Molecular-phylogenetic characterization of microbial community imbalances in human inflammatory bowel diseases. Proc. Natl. Acad. Sci. USA.

[11] Gibson G.R., Probert H.M., Loo J.V., Rastall R.A., Roberfroid M.B. (2004). Dietary modulation of the human colonic microbiota: Updating the concept of prebiotics. Nutr. Res. Rev..

[12] Cherbut C. (2002). Inulin and oligofructose in the dietary fibre concept. Br. J. Nutr..

[13] McRorie J.W. (2015). Psyllium is not fermented in the human gut. Neurogastroenterol. Motil..

[14] Puupponen-Pimiä R., Aura A.M., Oksman-Caldentey K.M., Myllärinen P., Saarela M., Mattila-Sandholm T., Poutenen K. (2002). Development of functional ingredients for gut health. Trends Food Sci. Technol..

[15] Weng C.J., Yen G.C. (2012). Chemopreventive effects of dietary phytochemicals against cancer invasion and metastasis: Phenolic acids, monophenol, polyphenol and their derivatives. Cancer Treat. Rev..

[16] Mursu J., Voutilainen S., Nurmi T., Tuomainen T.P., Kurt S., Salonen J.T. (2008). Flavonoid intake and the risk of ischaemic stroke and CVD mortality in middle-aged Finnish men: The kuopio ischemic heart disease risk factor study. Br. J. Nutr..

[17] Xia D., Wu X., Shi J., Yang Q., Zhang Y. (2011). Phenolic compounds from the edible seeds extract of Chinese Mei (Prunusmume Sieb. Et Zucc) and their antimicrobial activity. Food Sci. Technol..

[18] Lim S., Xu J., Kim J., Chen T.Y., Su X., Standard J., Carey E., Griffin J., Herndon B., Katz B. (2013). Role of anthocyanin-enriched purple-fleshed sweet potato p40 in colorectal cancer prevention. Mol. Nutr. Food Res..

[19] Han K.H., Matsumoto A., Shimada K., Sekikawa M., Fukushima M. (2007). Effects of anthocyanin-rich purple potato flakes on antioxidant status in F344 rats fed a cholesterol-rich diet. Br. J. Nutr..

[20] Jawi M., Wita W., Suprapta D.N. (2014). Extract of purple sweet potato tuber increases Sod and decreases VCAM-1 Expression by increasing Nrf2 expression in the aortic endothelia of hypercholesterolemic rabbits. J. Biol. Agric. Healthcare.

[21] Kano M., Takayanagi T., Harada K., Makino K., Ishikawa F. (2005). Antioxidative activity of anthocyanins from purple sweet potato, Ipomea batatas cultivar Ayamurasaki. Biosci. Biotechnol. Biochem..

[22] Han K.H., Sekikawa M., Shimada K., Hashimoto M., Hashimoto N., Noda T., Tanaka H., Fukushima M. (2006). Anthocyanin-rich purple potato flake extract has antioxidant capacity and improves antioxidant potential in rats. Br. J. Nutr..

[23] Singleton V.L., Orthofer R., Lamuela-Raventós R.M. (1999). Analysis of total polyphenol and other oxidation substrates and antioxidants by means of folin-ciocalteu reagent. Methods Enzymol..

[24] Nagata R., Echizen M., Yamaguchi Y., Han K.H., Shimada K., Ohba K., Kitano-Okada T., Nagura T., Uchino H., Fukushima M. (2018). Effect of a combination of inulin and polyphenol containing adzuki bean extract on intestinal fermentation In Vitro and In Vivo. Biosci. Biotechnol. Biochem..

[25] Yu Z., Morrison M. (2004). Improved extraction of PCR-quality community DNA from digesta and fecal samples. Biotechniques.

[26] Warren F.J., Fukuma N.M., Mikkelsen D., Flanagan B.M., Williams B.A., Lisle A.T., Cuív P.Ó., Morrison M., Gidley M.J. (2018). Food starch structure impacts gut microbiome composition. Msphere.

[27] Lozupone C., Knight R. (2005). UniFrac: A new phylogenetic method for comparing microbial communities. Appl. Environ. Microbiol..

[28] Zakrzewski M., Proietti C., Ellis J.J., Hasan S., Brion M.J., Berger B., Krause L. (2017). Calypso: A user-friendly web-server for mining and visualizing microbiome-environment interactions. Bioinformatics.

[29] Ikeda T., Tanaka Y., Yamamoto K., Morii H., Kamisako T., Ogawa H. (2014). Geranium Dielsianum extract powder (Miskamiska) improves the intestinal environment through alteration of microbiota and microbial metabolites in rats. J. Funct. Foods.

[30] Heinritz S.N., Rainer Mosenthin R., Weiss E. (2013). Use of pigs as a potential model for research into dietary modulation of the human gut microbiota. Nutr. Res. Rev..

[31] Schloss P.D., Westcott S.L., Ryabin T., Hall J.R., Hartmann M., Hollister E.B., Lesniewski R.A., Oakley B.B., Parks D.H., Robinson C.J. (2009). Introducing mothur: Open-Source, platform-independent, community-supported software for describing and comparing microbial communities. Appl. Environ. Microbiol..

[32] Frolinger T., Sims S., Smith C., Wang J., Cheng H., Faith J., Ho L., Hao K., Pasinetti G.M. (2019). The gut microbiota composition affects dietary polyphenols-mediated cognitive resilience in mice by modulating the bioavailability of phenolic acids. Sci. Rep..

[33] Espley R.V., Butts C.A., Laing W.A., Martell S., Smith H., McGhie T.K., Zhang J., Paturi G., Hedderley D., Bovy A. (2014). Dietary flavonoids from modified apple reduce inflammation markers and modulate gut microbiota in mice. J. Nutr..

[34] Kovatcheva-Datchary P., Nilsson A., Akrami R., Lee Y.S., De Vadder F., Arora T., Hallen A., Martens E., Björck I., Bäckhed F. (2015). Dietary fiber-induced improvement in glucose metabolism is associated with increased abundance of Prevotella. Cell Metab..

[35] Canard B., Cole S.T. (1989). Genome organization of the anaerobic pathogen Clostridium perfringens. Proc. Natl. Acad. Sci. USA..

[36] Koropatkin N.M., Cameron E.A., Martens E.C. (2012). How glycan metabolism shapes the human gut microbiota. Nat. Rev. Microbiol..

[37] Zhang X., Yang Y., Wu Z., Weng P. (2016). The modulatory effect of anthocyanins from purple sweet potato on human intestinal microbiota in vitro. J. Agric. Food Chem..

[38] Molan A.L., Liu Z., Kruger M. (2010). The ability of blackcurrant extracts to positively modulate key markers of gastrointestinal function in rats. World, J. Microbiol. Biotechnol..

[39] Scharlau D., Borowicki A., Habermann N., Hofmann T., Klenow S., Miene C., Munjal U., Stein K., Glei M. (2009). Mechanisms of primary cancer prevention by butyrate and other products formed during gut flora-mediated fermentation of dietary fibre. Mutat. Res..

[40] Crouse J.R., Gerson C.D., DeCarli L.M., Lieber C.S. (1968). Role of acetate in the reduction of plasma free fatty acids produced by ethanol in man. J. Lipid Res..

[41] Nastasi C., Candela M., Bonefeld C.M., Geisler C., Hansen M., Krejsgaard T., Biagi E., Andersen M.H., Brigidi P., θdum N. (2015). The effect of short-chain fatty acids on human monocyte-derived dendritic cells. Sci. Rep..

[42] Maeda T., Towatari M., Kosugi H., Saito H. (2000). Up-regulation of costimulatory/adhesion molecules by histone deacetylase inhibitors in acute myeloid leukemia cells. Blood.

[43] Kim H.J., White P.J. (2009). In vitro fermentation of oat flours from typical and high β-glucan oat lines. J. Agric. Food Chem..

[44] Zhu X.L., Zhang X., Sun Y.K., Su D., Sun Y., Hu B., Zeng X.X. (2013). Purification and fermentation in vitro of sesaminol triglucoside from sesame cake by human intestinal microbiota. J. Agric. Food Chem..

[45] Gibson G.R., Roberfroid M.B. (1995). Dietary modulation of the human colonic microbiota: Introducing the concept of prebiotics. J. Nutr..

[46] Davila A.M., Blachier F., Gotteland M., Andriamihaja M., Benetti P.H., Sanz Y., Tomé D. (2013). Intestinal luminal nitrogen metabolism: Role of the gut microbiota and consequences for the host. Parmacol. Res..

[47] Macfarlane G.T., Macfarlane S. (1997). Human colonic microbiota: Ecology, physiology and metabolic potential of intestinal bacteria. Scand. J. Gastroenterol. Suppl..

[48] Vanholder R., Schepers E., Pletinck A., Nagler E.V., Glorieux G. (2014). The uremic toxicity of indoxyl sulfate and p-cresyl sulfate: A systematic review. J. Am. Soc. Nephrol..

[49] Brocca A., Virzì G.M., de Cal M., Cantaluppi V., Ronco C. (2013). Cytotoxic effects of p-cresol in renal epithelial tubular cells. Blood Purif..

